# Spontaneous Visual Preference for Face-Like Stimuli Is Impaired in Newly-Hatched Domestic Chicks Exposed to Valproic Acid During Embryogenesis

**DOI:** 10.3389/fnbeh.2021.733140

**Published:** 2021-11-11

**Authors:** Alice Adiletta, Samantha Pedrana, Orsola Rosa-Salva, Paola Sgadò

**Affiliations:** Center for Mind/Brain Sciences, University of Trento, Rovereto, Italy

**Keywords:** autism spectrum disorder, face processing, social predispositions, brain development, sodium valproate

## Abstract

Faces convey a great amount of socially relevant information related to emotional and mental states, identity and intention. Processing of face information is a key mechanism for social and cognitive development, such that newborn babies are already tuned to recognize and orient to faces and simple schematic face-like patterns since the first hours of life. Similar to neonates, also non-human primates and domestic chicks have been shown to express orienting responses to faces and schematic face-like patterns. More importantly, existing studies have hypothesized that early disturbances of these mechanisms represent one of the earliest biomarker of social deficits in autism spectrum disorders (ASD). We used VPA exposure to induce neurodevelopmental changes associated with ASD in domestic chicks and tested whether VPA could impact the expression of the animals’ approach responses to schematic face-like stimuli. We found that VPA impairs the chicks’ preference responses to these social stimuli. Based on the results shown here and on previous studies, we propose the domestic chick as animal model to investigate the biological mechanisms underlying face processing deficits in ASD.

## Introduction

Biological predispositions to orient to and preferentially learn about conspecifics are one of the earliest expressions of social behavior in vertebrates and are critical for survival. These elementary behavioral markers of social orienting are spontaneous, possibly hard-wired, mechanisms that bias visual attention to simple features of animate beings since the earliest minutes of life (Goren et al., 1975; Johnson et al., 1991). Human faces and schematic face-like patterns generate remarkable responses in typical developing neonates (Simion and Di Giorgio, 2015). More strikingly, the same abilities can be observed in newly-hatched chicks (Rosa-Salva et al., 2010; Rosa Salva et al., 2011) and visually naïve monkeys (Sugita, 2008, 2009). Other species have also been shown to respond to similar schematic configurations (Leopold and Rhodes, 2010), such that privileged face processing could be pervasive in vertebrates.

More importantly, it has been hypothesized that early disturbances of these social orienting mechanisms may be one of the earliest signs of social deficits in autism spectrum disorders (ASD) and might also contribute to the pathophysiology of these disorders by compromising, early on, the typical developmental trajectories of the social brain (Dawson et al., 2005; Johnson, 2005; Senju and Johnson, 2009; Johnson et al., 2015). In line with that, impairments in face and eye-gaze direction processing have been reported in infants at risk of ASD (Di Giorgio et al., 2016; Webb et al., 2017, for a critical discussion see also Jones and Klin, 2013; Shultz et al., 2018; Bradshaw et al., 2020).

Given the complexity of human social behavior and the limitations that human studies impose, animal models are instrumental in providing clues on the nature and origin of these crucial social orienting mechanisms and their role in atypical social development. Valproic acid (VPA) exposure has been extensively used in several animal models to reproduce ASD core symptoms (Bambini-Junior et al., 2014). Previous studies have shown that exposure to different doses of VPA during embryogenesis induces alterations of several aspects of social behavior in domestic chicks (Nishigori et al., 2013; Zachar et al., 2019). We used VPA exposure to induce neurodevelopmental changes associated with social deficits in domestic chicks and tested whether VPA could impact the expression of early approach responses to schematic face-like patterns. We found that VPA impairs the chicks’ preference responses to these social stimuli. Based on the results shown here, we propose the domestic chicks as elective animal models to study these early-emerging neurobehavioral markers and to investigate the biological mechanisms underlying face processing deficits in ASD.

## Materials and Methods

### Ethical Approval

All experiments were conducted according to the current Italian and European Community laws for the ethical treatment of animals. The experimental procedures were approved by the Ethical Committee of the University of Trento and licensed by the Italian Health Ministry (permit number 986/2016-PR).

### Embryo Injections

Fertilized eggs of domestic chicks (*Gallus gallus*), of the Ross 308 (Aviagen) strain, were obtained from a local commercial hatchery [Agricola Berica, Montegalda (VI), Italy]. Upon arrival the eggs were placed in the dark and incubated at 37.5°C and 60% relative humidity, with rocking. One week before the predicted date of hatching, on embryonic day 14 (E14), fertilized eggs were selected by a light test, before injection. Chick embryo injection was performed according to previous reports (Nishigori et al., 2013; Sgadò et al., 2018). Briefly, a small hole was made on the egg shell above the air sac, and 35 μmoles of VPA (Sodium Valproate, Sigma Aldrich) were administered to each fertilized egg, in a volume of 200 μl, by dropping the solution onto the chorioallantoic membrane (VPA group). Age-matched control eggs were injected using the same procedure with 200 μL of vehicle (double distilled injectable water; CTRL group). After sealing the hole with paper tape, eggs were placed back in the incubator until E18, when they were placed in a hatching incubator (FIEM srl, Italy). Hatching took place at a temperature of 37.7°C, with 72% humidity. The day of hatching was considered post-hatching day 0 (P0).

### Rearing Conditions

After hatching in darkness, 69 chicks (38 males and 31 females) were kept in the hatching incubator for 24 h before the experiment.

### Apparatus and Test Stimuli

**FIGURE 1:**
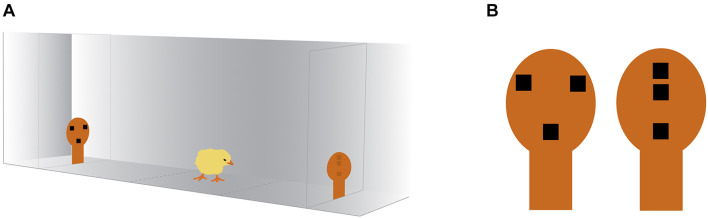
Schematic illustration of the social preference test apparatus and the stimuli. **(A)** The chick was placed in the center of the arena and was free to approach either of the stimuli, placed at the two ends of the apparatus and lit by a 201 lumen LED. The chick’s behavior was video-recorded from above. **(B)** The stimuli consisted of orange stiff paper silhouettes containing internal features resembling a face-like configuration (left) or a non-social control configuration (right). The chick image is courtesy of Openclipart (openclipart.org) under Creative Commons Zero 1.0 Public Domain License.

The test apparatus was a corridor, 45 cm long × 22.3 cm wide, made from wood and covered with opaque white plastic coating. The apparatus was divided in three sections (outlined on the apparatus floor), one central for positioning the animal, equidistant from the two stimuli, and two on the opposite side of the corridor, in proximity to the stimuli, considered the choice section. The stimuli were placed at the opposite side of the rectangular arena, on panels of light-filtering Plexiglas, lit by a 201 lumen LED placed behind the Plexiglas partition. The visual stimuli were previously described in Rosa-Salva et al. (2010). Briefly, they consisted of featureless face silhouette shapes, made of orange stiff paper (10 × 5.6 cm, see Figure 1) that contained internal features: three black squares (of side 1 cm), organized as an upside-down triangle for the schematic face-like configuration, or aligned vertically for the control non-social stimulus. Both stimuli were top-heavy configurations, having two elements in their upper part and one in their lower part.

### Test Procedures

At postnatal day 1 (P1), about 24 h after hatching, chicks were transported in complete darkness to the test room and placed in the apparatus: positioning with respect to the test stimuli, as well as the left-right position of the stimuli in the apparatus, was counterbalanced across animals. The animals’ approach responses were recorded using a camera placed on top of the apparatus, for the entire duration of the test (12 mins).

### Statistical Analysis

We evaluated the absolute time spent in each section of the apparatus (face section, central section, and non-face section) and the effect of treatment and sex on these measures, using a mixed model considering treatment and sex as fixed between-subject factors and the time spent in each apparatus section as fixed repeated measures (within subject factor with three levels: face section, central section, and non-face section). The relative preference expressed for the two stimuli was also measured as a social preference index adjusted for the overall exploratory activity of the chicks during the test. This was calculated as the time spent in the choice section close to the social stimulus (schematic face-like configuration) divided by the total time spent in the two choice sections (face + non-face). Values of this ratio range from 1 (full choice for the social stimulus) to 0 (full choice for the non-social stimulus), where 0.5 represents the absence of preference. Significant departures of the social preference index from chance level (0.5) were estimated by one-sample two-tailed *t*-tests. The number of chicks that first approached the two stimuli in the two treatment and sex groups was compared using two-sided Pearson’s chi-square test. We assessed differences in behavioral activity measuring the time required to move to one of the choice sections (latency to choice) and the number of section switches (spontaneous alternations). Effect of Treatment and Sex on the social preference index, the latency to first choice and the spontaneous alternations was evaluated by multifactorial analysis of variance (ANOVA). Statistical analyses were performed with GraphPad Prism 9 and RStudio. Alpha was set to 0.05 for all tests.

## Results

To assess the effect of VPA on face perception, and avoid any possible influence of previous experiences in evaluating the chicks’ approach to the stimuli, we excluded visual experience prior to the test. To obtain a better approach rate, we extended the duration of the test compared to the previous reports to 12 mins. Using this adapted paradigm, we tested 69 chicks (31 females, 38 males), 24 h after hatching.

**FIGURE 2:**
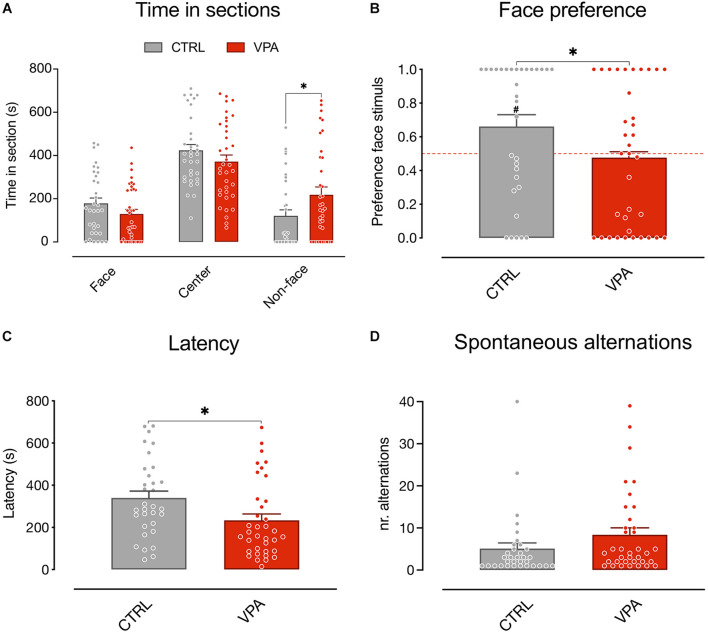
Spontaneous visual preference test. Social preference test for schematic face-like (social) stimulus and non-social stimulus (see “Materials and Methods” for details). Bar graphs represent time spent in the choice sections **(A)** social preferences indexes **(B)**, latency to first choice **(C)**, and spontaneous alternations **(D)**. **(A)** Mixed model analysis on the time spent in the three apparatus sections (face, center, and non-face) considering treatment and sex as fixed between subject factors and the time spent in each apparatus section as fixed repeated measures, shows a significant difference in the absolute time spent in the three sections (not shown) and a significant interaction between treatment and time spent in each apparatus section, and no other main effect or interactions between the factors analyzed. Sidak multiple comparison test shows a significant effect of treatment on the time spent in the non-face chamber. **(B)** Analysis of variance of social preference indexes using treatment and sex as between-subject factors, revealed a significant main effect of treatment and no other main effects or interactions among the factors analyzed. One-sample *t*-test on preference indexes indicate a significant difference from chance level for the control group, but not for VPA-treated chicks. The number sign (#) indicate significant departures of the preference index from chance level (0.5), marked by the red line. **(C)** Behavioral activity during the test measured as latency to *express a* choice. Analysis of variance on time taken by the chicks to move in one of the choice sections using treatment and sex as between-subject factors, showing a significant effect of treatment and no other main effects or interaction. **(D)** Behavioral activity during the test measured as *sections* alternations. Analysis of variance on number of *alternations* between the three *sections*, using treatment and sex as between-subject factors, showing no significant main effect of treatment or sex, and no interactions. Data represent Mean ± SEM, ^#^*p* < 0.05, ^∗^*p* < 0.05.

We first analyzed the time spent by the animals in the choice sections of the apparatus (Figure 2A) using a mixed model analysis (see “Materials and Methods”). The results showed no significant main effect of treatment and sex on the time spent in the apparatus sections [treatment *F*_(1, 195)_ = 4.812E-012, *p* > 0.9999; sex *F*_(1, 195)_ = 1.084E-010, *p* > 0.9999], a significant main effect of the sections [apparatus sections *F*_(2, 195)_ = 44.48, *p* < 0.0001] and a significant interaction of the treatment on the visited sections [treatment × apparatus sections *F*_(2, 195)_ = 4.904, *p* = 0.0084]. No other significant interactions emerged [treatment × sex *F*_(1, 195)_ = 9.114E-011, *p* > 0.9999; sex × apparatus sections *F*_(2, 195)_ = 0.4469, *p* = 0.6403; treatment × sex × apparatus sections *F*_(2, 195)_ = 1.287, *p* = 0.2784]. The Sidak multiple comparison test showed a significant effect of treatment on the time spent in the non-face chamber [*t*_(201)_ = 2.421, *p* = 0.0335]. Thus, VPA treatment selectively increases the time spent by the animals attending the non-face stimulus.

To further evaluate the effect of treatment on the preference for the stimuli independent of the exploratory activity we also analyzed the effect of VPA exposure on the preference index (see “Materials and Methods”). We found a significant difference between the treatment groups in the preference index for the schematic face-like configuration stimulus [Figure 2B; treatment: *F*_(1, 65)_ = 4.805, *p* = 0.0320; sex: *F*_(1, 65)_ = 0.5745, *p* = 0.4512; treatment × sex: *F*_(1, 65)_ = 2.652, *p* = 0.1083]. While vehicle-injected chicks significantly preferred the schematic face-like stimulus, VPA-exposed chicks did not display any significant preference for this stimulus compared to what expected by chance [Figure 2B; CTRL *t*_(32)_ = 2.481, *p* = 0.0186; VPA *t*_(35)_ = 0.3425, *p* = 0.7341; group mean: CTRL 0.6694 (95% CI: 0.5303–0.8085); VPA 0.4764 (95% CI: 0.3364–0.6164)]. We then analyzed the latency to express a choice and the number of section alternations after the first choice. We found a significant effect of treatment on the latency: VPA-injected chicks had a shorter latency to choice compared to controls [Figure 2C; treatment: *F*_(1, 65)_ = 5.369, *p* = 0.0237 sex: *F*_(1, 65)_ = 0.1881, *p* = 0.6660; treatment × sex: *F*_(1, 65)_ = 0.1270, *p* = 0.7228; group mean: CTRL 339 s (95% CI: 275–403); VPA 234 s (95% CI: 172–295)]. Spontaneous alternations in the two choice sections did not significantly differ between treatment groups [Figure 2D; treatment: *F*_(1, 65)_ = 1.941, *p* = 0.1683; sex: *F*_(1, 65)_ = 0.0790, *p* = 0.7795; treatment × sex: *F*_(1, 65)_ = 1.293, *p* = 0.2598; group mean: CTRL 5.091 (95% CI: 2.344–7.838); VPA 8.389 (95% CI: 5.077–11.70)].

The number of chicks that approached the face-like configuration as the first stimulus was not significantly different between treatment groups (Pearson’s *X*_1_^2^ = 2.944, *p* = 0.0862; CTRL: face *N* = 21, non-face *N* = 12, VPA: face *N* = 15, non-face *N* = 20, data not shown).

## Discussion

Newborns of several vertebrate species exhibit rudimental knowledge about the typical appearance of animate beings that orients the young organisms’ attention toward plausible social partners and caregivers. Several studies hypothesized that this mechanism contributes to create an early social bond with caretakers and social companions (Johnson, 2005; Tomalski et al., 2009), an essential process for subsequent social and language development. Newborn babies, as well as non-human primates and domestic chicks, have been shown to express remarkable orienting responses to faces and schematic face-like patterns (Sugita, 2008, 2009; Rosa-Salva et al., 2010; Rosa Salva et al., 2011). Divergence from these early social interactions may induce a cascade of maladaptive trajectories culminating in atypical social abilities, such as those observed in ASD.

Predispositions in domestic chicks have been observed toward a variety of features of animate creatures and trigger preference responses to a very broad spectrum of representations: being them face-like configurations (Rosa Salva et al., 2011) biological motion (Vallortigara et al., 2005) or self-propelled motion (Rosa-Salva et al., 2016). Newly hatched domestic chicks express social preferences to features of animals belonging to other species, including potential predators, as shown by their innate preference toward a walking cat (Vallortigara et al., 2005) represented by point light displays or toward a taxidermized polecat (Rosa-Salva et al., 2019) or a human face (Rosa Salva et al., 2011). Similarly, face-naïve Japanese macaques spend equal time attending to humans and monkey faces and prefer both over inanimate objects (Sugita, 2008). This data shows that biological predispositions are clearly not species-specific, but include rudimental configurations shared across species to increase the chance of orienting toward other animals. In the natural environment of a newly hatched organism, these other animals are most likely to be conspecifics (parents, siblings). Subsequently activated learning mechanisms, whose action is directed toward living creatures by the predispositions themselves, will provide the young animals with species-specific information on the appearance of their conspecifics [see also Morton and Johnson (1991) and Johnson (2005) for a broader discussion of the species-general nature of the representations underlying face-preferences in newborn babies and domestic chicks]. As to whether the face-like stimulus can be extended as a feature of conspecifics, studies show that the predisposed preference observed in newly-hatched chicks toward the stuffed hen or taxidermized newly-hatched chicks, mallard ducks or polecats, are indeed triggered by the head and neck region, suggesting a major role of face configurations in the head region (Johnson and Horn, 1988; Rosa-Salva et al., 2019; Miura et al., 2020).

Using the preference response to face-like stimuli as an evolutionarily conserved neurobehavioral marker and exploiting the advantages of animal models, we investigated whether these early-emerging social orienting mechanisms could be affected by a compound, VPA, known to interfere with development of the social brain. We examined the preference response toward schematic-face like configurations of animals whose pattern of brain development may have been altered by VPA, an anticonvulsant increasing the risk to develop ASD in humans. We found that VPA had a dramatic effect on the preference toward schematic-face configuration stimuli.

**FIGURE 3:**
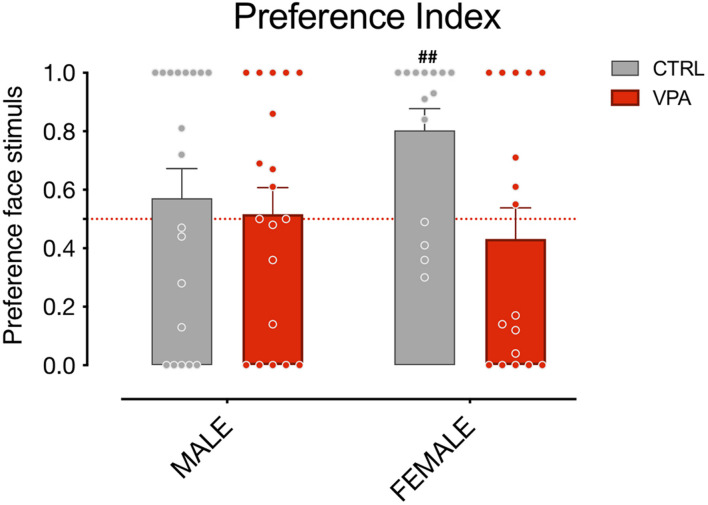
Social preference index of sex groups. Bar graphs represent social preference indexes for males and females. One-sample *t*-test on preference indexes indicate a significant difference from chance level for female control animals, but not for males or VPA-treated chicks of both sexes. The number sign (#) indicate significant departures of the preference index from chance level (0.5), marked by the red line. Data represents Mean ± SEM, ^##^uncorrected *p* < 0.01.

Previous studies have revealed a predisposed response to schematic face-like configurations in newly-hatched chicks, using both subjects imprinted on face-neutral stimuli and visually naïve subjects (Rosa-Salva et al., 2010; Rosa Salva et al., 2011, 2012). To assess the effect of VPA on face perception, and avoid any possible influence of previous experiences on the chicks’ approach to the stimuli, we applied this latter experimental procedure, excluding visual experience prior to the test. Since dark reared animals are less active compared to chicks exposed to visual stimuli, to obtain a better approach rate, we extended the duration of the test compared to the previous reports. Increasing the test duration in our experiment contributed to heighten the approach response and the face preference, without introducing the potential influence of visual experience. We also noticed that the preference for the face-like stimulus was especially conspicuous in control females, which showed a remarkable preference level compared to all other groups [Figure 3; group mean preference index CTRL females 0.8029 (95% CI: 0.6425–0.9632), one-sample *t*-test *t*_(13)_ = 4.081, uncorrected *p* = 0.0013; group mean preference index VPA females 0.4318 (95% CI: 0.2064–0.6571); *t*_(12)_ = 0.6419, uncorrected *p* = 0.5301; group mean preference index CTRL males 0.5711 (95% CI: 0.3589–0.7832); *t*_(18)_ = 0.7035, uncorrected *p* = 0.4907; group mean preference index VPA males 0.5163 (95% CI: 0.3245–0.7081); *t*_(18)_ = 0.1787, uncorrected *p* = 0.8602]. However, given that no significant interaction between the factors sex and treatment emerged in our previous analysis, any difference between the two sexes observed here should be interpreted with caution. Notably, regardless of the sex of the chicks examined, VPA-exposed chicks spent significantly more time attending the non-social stimulus. This data is in line with what observed in other VPA models (Zhao et al., 2019) in which juvenile VPA-treated monkeys attended to non-social stimuli significantly more than their control siblings. Future studies will investigate the potential sex differences in the level of face-preference and in their susceptibility to VPA, suggested by some of our data, and clarify the mechanism of action of VPA on the development and expression of face preference in domestic chicks.

The reduced latencies observed in the VPA group, indicate that VPA exposure affects the visual preference for schematic face-configuration patterns without significantly hindering the chicks’ motoric activity during the test. In line with that, previous studies from our lab have shown that VPA exposure, at the dosage used in this study, does not significantly affect motor behavior or discriminative abilities of simple artificial objects in domestic chicks (Sgadò et al., 2018).

A previous study has investigated the attentive behavior toward faces in VPA-exposed juvenile macaques (Zhao et al., 2019). Using eye-tracking analysis to measure the animals’ attention to faces or scene containing conspecifics, the authors found that juvenile VPA-treated monkeys attended to non-social stimuli significantly more than their control siblings. However, the study did not specifically investigate the predisposed response of visually naïve animals to faces compared to a visually equivalent stimulus without social content. In this respect, our study is the first to analyze a very early predisposed response to faces in a visually naïve animal model of ASD.

Valproic acid is an anticonvulsant extensively used to treat epilepsy and bipolar disorders. VPA mechanism of action involves its direct inhibition of histone deacetylases (HDACs), interfering with normal deacetylation of chromatin and disrupting gene transcription at global scale, as well as HDAC independent mechanisms (Sinha et al., 2021). Embryonic exposure to VPA is normally achieved by a single acute dose of VPA (ranging between 400 and 800 mg/kg in rodents) that induces a transient HDAC inhibition producing long lasting effects. Several studies suggest that embryonic VPA exposure affects neurogenesis (Kataoka et al., 2013; Lee et al., 2016; Sakai et al., 2018; Zhao et al., 2019; Cui et al., 2020; Sawada et al., 2021) and alters expression of several neurodevelopmental genes, involving serotonergic system development (Jacob et al., 2014; Messina et al., 2020) and excitation/inhibition imbalance (Rinaldi et al., 2007; Gogolla et al., 2009; Banerjee et al., 2013; Nagode et al., 2017). Given its antiepileptic pharmacological action, VPA has been shown to increase GABA levels in the brain, trough different mechanisms, acting on GABA transaminase and other enzymes linked to the metabolism of GABA (Johannessen, 2000), as well as trough inhibition of sodium channels (Abdelsayed and Sokolov, 2013). Despite extensive research investigating VPA pharmacological action and the genetic networks responsible for its effects on brain development, the biological mechanisms underlying the detrimental consequences of embryonic VPA exposure on social behavior in animal models are still unclear.

## Conclusion

Altogether, this study and previous studies from our lab, demonstrate a detrimental effect of VPA, an anticonvulsant increasing the risk to develop ASD in humans, on the very early predisposed responses toward social stimuli in visually-naïve domestic chicks. Based on these results, we propose the domestic chicks as elective animal models to study these early-emerging neurobehavioral markers and to investigate the biological mechanisms underlying face processing deficits in ASD.

## Data Availability Statement

The original contributions presented in the study are included in the article/Supplementary Material, further inquiries can be directed to the corresponding author.

## Ethics Statement

The animal study was reviewed and approved by Ethical Committee of the University of Trento and licensed by the Italian Health Ministry (permit number 986/2016-PR).

## Author Contributions

PS conceived and designed the experiments and drafted the manuscript. AA and SP conducted the experiments. PS and OR-S analyzed the data. AA, PS, and OR-S wrote the manuscript. All authors read and approved the final manuscript.

## Conflict of Interest

The authors declare that the research was conducted in the absence of any commercial or financial relationships that could be construed as a potential conflict of interest.

## Publisher’s Note

All claims expressed in this article are solely those of the authors and do not necessarily represent those of their affiliated organizations, or those of the publisher, the editors and the reviewers. Any product that may be evaluated in this article, or claim that may be made by its manufacturer, is not guaranteed or endorsed by the publisher.

## References

[B1] Abdelsayed M. Sokolov S. (2013). Voltage-gated sodium channels: pharmaceutical targets via anticonvulsants to treat epileptic syndromes. *Channels* 7 146–152. 10.4161/chan.24380 23531742 PMC3710341

[B2] Bambini-Junior V. Baronio D. Mackenzie J. Zanatta G. Riesgo R. D. S. Gottfried C. (2014). “Prenatal exposure to valproate in animals and autism,” in *Comprehensive Guide to Autism*, eds Patel V. Preedy V. Martin C. (New York, NY: Springer New York), 1779–1793.

[B3] Banerjee A. García-Oscos F. Roychowdhury S. Galindo L. C. Hall S. Kilgard M. P. (2013). Impairment of cortical GABAergic synaptic transmission in an environmental rat model of autism. *Int. J. Neuropsychopharmacol.* 16 1309–1318. 10.1017/S1461145712001216 23228615 PMC3674140

[B4] Bradshaw J. Klin A. Evans L. Klaiman C. Saulnier C. Mccracken C. (2020). Development of attention from birth to 5 months in infants at risk for autism spectrum disorder. *Dev. Psychopathol.* 32 491–501. 10.1017/S0954579419000233 31012398 PMC6812597

[B5] Cui K. Wang Y. Zhu Y. Tao T. Yin F. Guo Y. (2020). Neurodevelopmental impairment induced by prenatal valproic acid exposure shown with the human cortical organoid-on-a-chip model. *Microsyst. Nanoeng.* 6:49. 10.1038/s41378-020-0165-z 34567661 PMC8433196

[B6] Dawson G. Webb S. J. Mcpartland J. (2005). Understanding the nature of face processing impairment in autism: insights from behavioral and electrophysiological studies. *Dev. Neuropsychol.* 27 403–424. 10.1207/s15326942dn2703_6 15843104

[B7] Di Giorgio E. Frasnelli E. Rosa Salva O. Scattoni M. L. Puopolo M. Tosoni D. (2016). Difference in visual social predispositions between newborns at low- and high-risk for autism. *Sci. Rep.* 6:26395. 10.1038/srep26395 27198160 PMC4873740

[B8] Gogolla N. Leblanc J. J. Quast K. B. Südhof T. C. Fagiolini M. Hensch T. K. (2009). Common circuit defect of excitatory-inhibitory balance in mouse models of autism. *J. Neurodev. Disord.* 1 172–181. 20664807 10.1007/s11689-009-9023-x PMC2906812

[B9] Goren C. C. Sarty M. Wu P. Y. (1975). Visual following and pattern discrimination of face-like stimuli by newborn infants. *Pediatrics* 56 544–549. 1165958

[B10] Jacob J. Ribes V. Moore S. Constable S. C. Sasai N. Gerety S. S. (2014). Valproic acid silencing of ascl1b/Ascl1 results in the failure of serotonergic differentiation in a *zebrafish* model of fetal valproate syndrome. *Dis. Model Mech.* 7 107–117. 10.1242/dmm.013219 24135485 PMC3882053

[B11] Johannessen C. U. (2000). Mechanisms of action of valproate: a commentatory. *Neurochem. Int.* 37 103–110. 10.1016/s0197-0186(00)00013-9 10812195

[B12] Johnson M. H. (2005). Subcortical face processing. *Nat. Rev. Neurosci.* 6 766–774. 10.1038/nrn1766 16276354

[B13] Johnson M. H. Dziurawiec S. Ellis H. Morton J. (1991). Newborns’ preferential tracking of face-like stimuli and its subsequent decline. *Cognition* 40 1–19. 10.1016/0010-0277(91)90045-6 1786670

[B14] Johnson M. H. Horn G. (1988). Development of filial preferences in dark-reared chicks. *Anim. Behav.* 36 675–683. 10.1016/S0003-3472(88)80150-7

[B15] Johnson M. H. Senju A. Tomalski P. (2015). The two-process theory of face processing: modifications based on two decades of data from infants and adults. *Neurosci. Biobehav. Rev.* 50 169–179. 10.1016/j.neubiorev.2014.10.009 25454353

[B16] Jones W. Klin A. (2013). Attention to eyes is present but in decline in 2-6-month-old infants later diagnosed with autism. *Nature* 504 427–431. 10.1038/nature12715 24196715 PMC4035120

[B17] Kataoka S. Takuma K. Hara Y. Maeda Y. Ago Y. Matsuda T. (2013). Autism-like behaviours with transient histone hyperacetylation in mice treated prenatally with valproic acid. *Int. J. Neuropsychopharmacol.* 16 91–103. 10.1017/S1461145711001714 22093185

[B18] Lee H. J. Dreyfus C. Dicicco-Bloom E. (2016). Valproic acid stimulates proliferation of glial precursors during cortical gliogenesis in developing rat. *Dev. Neurobiol.* 76 780–798. 10.1002/dneu.22359 26505176

[B19] Leopold D. A. Rhodes G. (2010). A comparative view of face perception. *J. Comp. Psychol.* 124 233–251. 10.1037/a0019460 20695655 PMC2998394

[B20] Messina A. Boiti A. Sovrano V. A. Sgadò P. (2020). Micromolar valproic acid doses preserve survival and induce molecular alterations in neurodevelopmental genes in two strains of *zebrafish* larvae. *Biomolecules* 10:1364. 10.3390/biom10101364 PMC7601180 32987891

[B21] Miura M. Nishi D. Matsushima T. (2020). Combined predisposed preferences for colour and biological motion make robust development of social attachment through imprinting. *Anim. Cogn.* 23 169–188. 10.1007/s10071-019-01327-5 31712936

[B22] Morton J. Johnson M. H. (1991). CONSPEC and CONLERN: a two-process theory of infant face recognition. *Psychol. Rev.* 98 164–181. 2047512 10.1037/0033-295x.98.2.164

[B23] Nagode D. A. Meng X. Winkowski D. E. Smith E. Khan-Tareen H. Kareddy V. (2017). Abnormal development of the earliest cortical circuits in a mouse model of autism spectrum disorder. *Cell Rep.* 18 1100–1108. 10.1016/j.celrep.2017.01.006 28147267 PMC5488290

[B24] Nishigori H. Kagami K. Takahashi A. Tezuka Y. Sanbe A. Nishigori H. (2013). Impaired social behavior in chicks exposed to sodium valproate during the last week of embryogenesis. *Psychopharmacology* 227 393–402. 10.1007/s00213-013-2979-y 23371491

[B25] Rinaldi T. Kulangara K. Antoniello K. Markram H. (2007). Elevated NMDA receptor levels and enhanced postsynaptic long-term potentiation induced by prenatal exposure to valproic acid. *Proc. Natl. Acad. Sci. U. S. A.* 104 13501–13506. 10.1073/pnas.0704391104 17675408 PMC1948920

[B26] Rosa Salva O. Farroni T. Regolin L. Vallortigara G. Johnson M. H. (2011). The evolution of social orienting: evidence from chicks (*Gallus gallus*) and human newborns. *PLoS One* 6:e18802. 10.1371/journal.pone.0018802 21533093 PMC3080385

[B27] Rosa Salva O. Regolin L. Vallortigara G. (2012). Inversion of contrast polarity abolishes spontaneous preferences for face-like stimuli in newborn chicks. *Behav. Brain Res.* 228 133–143. 10.1016/j.bbr.2011.11.025 22155610

[B28] Rosa-Salva O. Grassi M. Lorenzi E. Regolin L. Vallortigara G. (2016). Spontaneous preference for visual cues of animacy in naïve domestic chicks: the case of speed changes. *Cognition* 157 49–60. 10.1016/j.cognition.2016.08.014 27592411

[B29] Rosa-Salva O. Mayer U. Vallortigara G. (2019). Unlearned visual preferences for the head region in domestic chicks. *PLoS One* 14:e0222079. 10.1371/journal.pone.0222079 31479480 PMC6719852

[B30] Rosa-Salva O. Regolin L. Vallortigara G. (2010). Faces are special for newly hatched chicks: evidence for inborn domain-specific mechanisms underlying spontaneous preferences for face-like stimuli. *Dev. Sci.* 13 565–577. 10.1111/j.1467-7687.2009.00914.x 20590721

[B31] Sakai A. Matsuda T. Doi H. Nagaishi Y. Kato K. Nakashima K. (2018). Ectopic neurogenesis induced by prenatal antiepileptic drug exposure augments seizure susceptibility in adult mice. *Proc. Natl. Acad. Sci. U. S. A.* 115 4270–4275. 10.1073/pnas.1716479115 29610328 PMC5910824

[B32] Sawada K. Kamiya S. Aoki I. (2021). Neonatal valproic acid exposure produces altered gyrification related to increased parvalbumin-immunopositive neuron density with thickened sulcal floors. *PLoS One* 16:e0250262. 10.1371/journal.pone.0250262 33878144 PMC8057614

[B33] Senju A. Johnson M. H. (2009). Atypical eye contact in autism: models, mechanisms and development. *Neurosci. Biobehav. Rev.* 33 1204–1214. 10.1016/j.neubiorev.2009.06.001 19538990

[B34] Sgadò P. Rosa-Salva O. Versace E. Vallortigara G. (2018). Embryonic exposure to valproic acid impairs social predispositions of newly-hatched chicks. *Sci. Rep.* 8:5919. 10.1038/s41598-018-24202-8 29650996 PMC5897402

[B35] Shultz S. Klin A. Jones W. (2018). Neonatal transitions in social behavior and their implications for autism. *Trends Cogn. Sci.* 22 452–469. 10.1016/j.tics.2018.02.012 29609895 PMC6554740

[B36] Simion F. Di Giorgio E. (2015). Face perception and processing in early infancy: inborn predispositions and developmental changes. *Front. Psychol.* 6:969. 10.3389/fpsyg.2015.00969 26217285 PMC4496551

[B37] Sinha P. Cree S. L. Miller A. L. Pearson J. F. Kennedy M. A. (2021). Transcriptional analysis of sodium valproate in a serotonergic cell line reveals gene regulation through both HDAC inhibition-dependent and independent mechanisms. *Pharmacogenomics J.* 21 359–375. 10.1038/s41397-021-00215-x 33649518

[B38] Sugita Y. (2008). Face perception in monkeys reared with no exposure to faces. *Proc. Natl. Acad. Sci. U. S. A.* 105 394–398. 10.1073/pnas.0706079105 18172214 PMC2224224

[B39] Sugita Y. (2009). Innate face processing. *Curr. Opin. Neurobiol.* 19 39–44. 10.1016/j.conb.2009.03.001 19339171

[B40] Tomalski P. Csibra G. Johnson M. H. (2009). Rapid orienting toward face-like stimuli with gaze-relevant contrast information. *Perception* 38 569–578. 10.1068/p6137 19522324

[B41] Vallortigara G. Regolin L. Marconato F. (2005). Visually inexperienced chicks exhibit spontaneous preference for biological motion patterns. *PLoS Biol.* 3:e208. 10.1371/journal.pbio.0030208 15934787 PMC1150290

[B42] Webb S. J. Neuhaus E. Faja S. (2017). Face perception and learning in autism spectrum disorders. *Q. J. Exp. Psychol.* 70 970–986. 10.1080/17470218.2016.1151059 26886246 PMC5026554

[B43] Zachar G. Tóth A. S. Gerecsei L. I. Zsebők S. Ádám Á Csillag A. (2019). Valproate exposure in ovo attenuates the acquisition of social preferences of young post-hatch domestic chicks. *Front. Physiol.* 10:881. 10.3389/fphys.2019.00881 31379596 PMC6646517

[B44] Zhao H. Wang Q. Yan T. Zhang Y. Xu H. J. Yu H. P. (2019). Maternal valproic acid exposure leads to neurogenesis defects and autism-like behaviors in non-human primates. *Transl. Psychiatry* 9:267. 10.1038/s41398-019-0608-1 31636273 PMC6803711

